# Reconfiguration of metabolic fluxes in *Pseudomonas putida* as a response to sub-lethal oxidative stress

**DOI:** 10.1038/s41396-020-00884-9

**Published:** 2021-01-11

**Authors:** Pablo I. Nikel, Tobias Fuhrer, Max Chavarría, Alberto Sánchez-Pascuala, Uwe Sauer, Víctor de Lorenzo

**Affiliations:** 1 The Novo Nordisk Foundation Center for Biosustainability, Technical University of Denmark, 2800 Kgs Lyngby, Denmark; 2 Institute of Molecular Systems Biology, ETH Zurich, 8093 Zurich, Switzerland; 3 Escuela de Química & CIPRONA, Universidad de Costa Rica, 2060 San José, Costa Rica; 4 Department of Biochemistry and Synthetic Metabolism, Max Planck Institute for Terrestrial Microbiology, 35043 Marburg, Germany; 5 Systems and Synthetic Biology Program, Centro Nacional de Biotecnología (CNB-CSIC), 28049 Madrid, Spain

**Keywords:** Bacteriology, Environmental sciences

## Abstract

As a frequent inhabitant of sites polluted with toxic chemicals, the soil bacterium and plant-root colonizer *Pseudomonas putida* can tolerate high levels of endogenous and exogenous oxidative stress. Yet, the ultimate reason of such phenotypic property remains largely unknown. To shed light on this question, metabolic network-wide routes for NADPH generation—the metabolic currency that fuels redox-stress quenching mechanisms—were inspected when *P*. *putida* KT2440 was challenged with a sub-lethal H_2_O_2_ dose as a proxy of oxidative conditions. ^13^C-tracer experiments, metabolomics, and flux analysis, together with the assessment of physiological parameters and measurement of enzymatic activities, revealed a substantial flux reconfiguration in oxidative environments. In particular, periplasmic glucose processing was rerouted to cytoplasmic oxidation, and the cyclic operation of the pentose phosphate pathway led to significant NADPH-forming fluxes, exceeding biosynthetic demands by ~50%. The resulting NADPH surplus, in turn, fueled the glutathione system for H_2_O_2_ reduction. These properties not only account for the tolerance of *P. putida* to environmental insults—some of which end up in the formation of reactive oxygen species—but they also highlight the value of this bacterial host as a platform for environmental bioremediation and metabolic engineering.

## Introduction

Environmental bacteria are exposed to different types of stress in the niches they colonize. *Pseudomonas putida* is an archetypal instance of a strictly aerobic, Gram-negative soil bacterium characterized by a remarkable metabolic versatility [1–4], and equipped with the enzymatic machinery needed to catabolize both natural and recalcitrant aromatic compounds besides sugars and organic acids [5]. The metabolic pathways deployed for biodegradation of organic and xenobiotic chemicals [6] often involve harsh redox reactions prone to generate reactive oxygen species (ROS) [7]. Apart of this endogenous source of stress, many of the scenarios where bacteria meet environmental pollutants are characterized by the presence of both direct and indirect oxidative agents. Besides the presence of oxygen in the upper soil layers [8], cycles of inundation, elevation gradients, salinity fluctuations, sunlight, and other abiotic factors can generate ROS—thereby leading to an oxidative milieu [9–11]. Environmental bacteria have evolutionary acquired mechanisms to cope with such conditions and, besides *Pseudomonas*, species of the *Burkholderia* [12] and *Sphingomonas* [13] genera—typically used in bioaugmentation—are recognized to withstand harsh physicochemical conditions. Yet, how do these environmental microorganisms cope with redox insults? Peroxides and free radicals damage virtually all macromolecular components of the cell (e.g., proteins, lipids, and DNA [14, 15]), and mechanisms to prevent (or repair) the damage exerted by ROS are key components of virtually all biological systems [16]. Long-term transcriptional responses that lead to mechanisms of ROS scavenging, for instance, appear to be conserved across species [17]. In *Escherichia coli*, for instance, oxidative challenges cause upregulation of genes encoding ROS-quenching superoxide dismutases, catalase, and glutathione/glutaredoxin recycling systems [18–21]. However, until transcriptionally controlled defense mechanisms become fully operational, cell survival depends on the default activity of the above-mentioned enzymes and the availability of antioxidants (e.g. reduced glutathione [22, 23]) to scavenge ROS generated by endogenous reactions or external oxidative damage. Prior to full-fledged deployment of transcriptional responses (which can take from several minutes up to hours, depending on the species and the growth conditions [24, 25]), metabolic reactions are the first line of defense upon sudden exposure to oxidative stress [26–29], as long as they can provide reducing power to fuel ROS-detoxifying enzymes [30]. The specific metabolic mechanisms that underlay the tolerance of *P. putida* to oxidative stress, however, remain largely unknown.

The adaptability of *P*. *putida* KT2440 (type strain of this species [31]) to adverse conditions, including redox stress, is wired to a unique metabolic architecture. Glucose catabolism in *P*. *putida* relies on the Entner–Doudoroff (ED) pathway [32], which starts with 6-phosphogluconate (6PG) as the substrate, formed via separate and converging routes for hexose phosphorylation (in the cytoplasm) or oxidation (in the periplasm) [33, 34]. Additionally, the *EDEMP cycle*, a combination of enzymes from the ED, the pentose phosphate (PP) and the (incomplete) Embden–Meyerhof–Parnas (EMP) pathways [35], processes hexoses phosphate to feed the lower catabolism, i.e. downwards phosphoenolpyruvate (PEP), pyruvate (Pyr), and the tricarboxylic acid (TCA) cycle. In addition to catabolic processing of sugars, the operation of the EDEMP cycle entails redirecting part of the trioses phosphate back to hexoses phosphate via the gluconeogenic activity of the EMP branch of the cycle [36]. As such, cyclic sugar catabolism contributes to a slight catabolic overproduction of NADPH in glucose cultures [35]. Although mostly studied with model carbon sources, the EDEMP cycle is the authentic metabolic heart of *P. putida* and is key for the hierarchical consumption of sugars and aromatic compounds [37], also under fluctuating conditions of iron availability [38]. While the main sink of NADPH is the anabolic use of reducing power for biomass formation, this redox cofactor is the major metabolic currency to counterfeit oxidative stress, since it serves as the reductant for several ROS-detoxifying enzymes and mechanisms [39], e.g. glutathione regeneration. However, the connection between the operation of central carbon metabolism and oxidative stress responses have remained elusive thus far.

In this work, the reshaping of the central metabolism in *P*. *putida* KT2440 in response to a sub-lethal oxidative stress levels, elicited by exposure to hydrogen peroxide (H_2_O_2_), has been thoroughly inspected. The strategy encompassed ^13^C-tracer experiments, metabolomics, and metabolic flux analysis, combined with the determination of physiological parameters, in vitro measurement of key enzymatic activities and assessment of glutathione-mediated ROS-detoxifying mechanisms. The results revealed adaptions in the operation of the EDEMP cycle, concomitant with a decrease in the flux through sugar oxidation pathways for glucose processing, which helps replenishing the NADPH pool upon oxidative challenges. These metabolic adjustments are discussed at the light of the lifestyle of *P*. *putida* (and related environmental bacteria) and the value of this species as robust agent for both in situ remediation of environmental pollution and metabolic engineering.

## Materials and methods

### Bacterial strain and culture conditions

Wild-type *P*. *putida* strain KT2440 was used throughout this study [40], and it was routinely grown in LB medium [41]. Quantitative physiology experiments were carried out in M9 minimal medium containing 6 g L^−1^ Na_2_HPO_4_, 3 g L^−1^ KH_2_PO_4_, 1.4 g L^−1^ (NH_4_)_2_SO_4_, 0.5 g L^−1^ NaCl, 0.2 g L^−1^ MgSO_4_·7H_2_O and 2.5 mL L^−1^ of a trace elements solution [42]. Unless noted otherwise, either natural or ^13^C-labeled glucose was used as the sole carbon source at 20 mM. Growth was estimated by measuring the optical density at 600 nm (OD_600_) after diluting the culture with 9 g L^−1^ NaCl. Correlation factors between cell dry weight (CDW) and OD_600_ were determined in batch cultures [43]. All cultures were started with an isolated colony from an LB medium plate, suspended in 5 mL of M9 minimal medium in a test tube. After an 18-h incubation, this culture was used to inoculate fresh medium at an OD_600_ of 0.01. Working cultures were cultivated in 250-mL Erlenmeyer flasks containing medium up to one-fifth of their nominal volume. H_2_O_2_ was used as an oxidative stress agent at 1.5 mM, added at OD_600_ = 0.5 (mid-exponential phase). Cultures were incubated for an additional 1.5 h, and samples were taken for analyses as explained below.

### Determination of physiological parameters

Regression analysis was applied during exponential growth to calculate [i] the maximum specific growth rate (μ), [ii] the biomass yield on substrate (*Y*_X/S_), [iii] the specific rate of glucose consumption (*q*_S_), and [iv] the molar yield of organic acids on glucose (*y*_P/S_). CDW was quantified by harvesting cells by fast filtration in pre-weighed nitrocellulose filters (0.45 μm pore), washed twice with 9 g L^−1^ NaCl, and dried at 105 °C to a constant weight. Glucose was assayed using a commercial kit from R-Biopharm AG (Darmstadt, Germany). The concentration of gluconate and 2-ketogluconate in culture supernatants was measured enzymatically [44]. In order to assess the impact of different carbon sources in the growth phenotype of strain KT2440 upon oxidative challenges, batch cultures were developed in M9 minimal medium as indicated above, and either glucose (20 mM), α-ketoglutarate (24 mM), or glycerol (40 mM) were used as the sole carbon substrate. Note that these culture conditions provide a total carbon concentration of 120 mM. When the OD_600_ of the cultures reached 0.15, H_2_O_2_ was added at 3 mM, and the change in OD_600_ was monitored every 15 min until the stationary phase was reached. Normalized growth coefficients (i.e., the ratio between μ in stressed cultures and μ in control experiments) were calculated for each culture condition [45].

### Determination of relative metabolite concentrations and ^13^C-labeling patterns by LC-MS/MS

Cultures were grown on either 100% [1-^13^C_1_]-glucose or 100% [6-^13^C_1_]-glucose as the sole carbon source. The biomass corresponding to 0.5–0.6 mg of CDW was collected in triplicates by fast centrifugation (13,000 × *g*, 30 s, −4 °C). Bacterial pellets were immediately frozen in liquid N_2_. Samples were then extracted three times with 0.5 mL of 60% (v/v) ethanol buffered with 10 mM ammonium acetate (pH = 7.2) at 78 °C for 1 min. After each extraction step, the biomass was separated by centrifugation at 13,000 × *g* for 1 min. The three liquid extracts were pooled and dried at 120 μbar, and stored at −80 °C thereafter. Samples were resuspended in 20 μL of MilliQ water, distributed in sealed 96-well microtiter plates, and injected into a Waters Acquity UPLC (Waters Corp., Milford, MA, USA) with a Waters Acquity T3 column (150 mm × 2.1 mm × 1.8 μm, Waters Corp.) coupled to a Thermo TSQ Quantum Ultra triple quadrupole instrument (Thermo Fisher Scientific Inc., Waltham, MA) with electrospray ionization. ^13^C-Labeling patterns of free intracellular metabolites were determined for dihydroxyacetone phosphate, fructose-6-phosphate (F6P), fructose-1,6-bisphosphate (FBP), glucose-6-phosphate (G6P), 6PG, PEP, ribose-5-phosphate (R5P), ribulose-5-phosphate (Ru5P), sedoheptulose-7-phosphate (S7P), xylulose-5-phosphate, and Pyr as described previously [46]. All other metabolites considered for the analysis are listed in Dataset S1. Relative abundances were calculated as the total ion count of all measured ^13^C isotopes for each metabolite across the different growth conditions, and presented as ratios whenever relevant. The raw data of labeling experiments is available in Dataset S1.

### Determination of ^13^C-labeling patterns by GC-MS

Cultures were grown on either 100% [1-^13^C_1_]-glucose or a mixture of 20% (w/w) [U-^13^C_6_]-glucose and 80% (w/w) naturally labeled glucose, and 5 mL aliquots of cell broth were harvested by centrifugation at 1,200 × *g* and −4 °C for 10 min. Bacterial pellets were washed twice with 1 mL of 9 g L^−1^ NaCl, hydrolyzed in 1 mL of 6 M HCl for 24 h at 110 °C, and desiccated overnight at 85 °C under a constant air stream. The hydrolyzate was dissolved in 50 μL of 99.8% (w/v) dimethyl formamide and subsequently transferred into a new tube. For sample derivatization, 30 μL of *N*-methyl-*N*-(*tert*-butyldimethylsilyl)-trifluoroacetamide was added to the biomass hydrolyzate and incubated at 85 °C for 60 min. The ^13^C-labeling patterns of proteinogenic amino acids were determined on a 6890 N Network GC system with a 5975 inert XL mass selective detector (Agilent Technologies Inc., Santa Clara, CA, USA) [47, 48]. The raw GC-MS data from four independent experiments is presented in Dataset S2.

### Metabolic flux ratio analysis

Mass distribution vectors of the proteinogenic amino acids were corrected for the natural abundance of all stable isotopes, and the relative metabolic flux ratios [i] oxaloacetate (OAA) from Pyr, [ii] glyoxylate shunt, [iii] PEP from OAA, and [iv] the lower and upper bound for Pyr from malate were calculated using the Fiat Flux software [49]. The mass distribution vectors of the free intracellular metabolites were corrected for the natural abundance of all stable isotopes using MatLab (The Mathworks Inc., Natick, MA, USA). Novel relative flux ratios were defined and calculated as described by Nikel et al. [35]. The fraction of G6P originating from glucose was estimated using data from the experiments using 100% [1-^13^C_1_]-glucose, whereas 6PG from G6P was calculated using data from either 100% [1-^13^C_1_]-glucose or 100% [6-^13^C_1_]-glucose experiments. Formation of F6P through the PP pathway was estimated using data from 100% [6-^13^C_1_]-glucose experiments, and Pyr through the ED pathway was assessed with data from 100% [1-^13^C_1_]-glucose experiments. The metabolic model used for net-flux analysis was based on a master reaction network with 45 reactions and 33 metabolites. Fluxes were calculated using [i] the stoichiometric reaction matrix, [ii] constraints accounting for the ratios from FiatFlux analysis and additionally for the ratios in the initial steps of glucose catabolism as described above, [iii] physiological data, and [iv] precursor requirements for biomass. The experimentally determined relative flux ratios were translated into constraints as follows (the reaction numbers, *v*_x_, are defined in Fig. S1). The fraction of G6P originating from glucose (*a*) was estimated as *a* = *v*_3_/(*v*_3_ + *v*_16_); the fraction of F6P originating through the PP pathway (*b*) was calculated as *b* = (*v*_14_ + *v*_15_)/(*v*_14_ + *v*_15_ + *v*_17_); and the fraction of 6PG originating from G6P (*c*) was determined as *c* = *v*_7_/(*v*_4_ + *v*_6_ + *v*_7_). For this ratio, the values from the experiments using data from either 100% [1-^13^C_1_]-glucose or [6-^13^C_1_]-glucose were averaged. The fraction of Pyr originating through the ED pathway (*d*) was determined as *d* = *v*_9_/(*v*_9_ + *v*_22_ + *v*_33_ + *v*_34_). The upper and lower bounds of Pyr originating from malate (*e* and *f*, respectively) were obtained according to *e* ≥ *v*_34_/(*v*_9_ + *v*_22_ + *v*_33_ + *v*_34_) and *f* ≤ *v*_34_/(*v*_9_ + *v*_22_ + *v*_33_ + *v*_34_). The fraction of OAA originating from Pyr (*g*) was obtained following *g* = *v*_32_/(*v*_30_ + *v*_32_). Finally, the fraction of Pyr originating from OAA (*h*) was derived from *h* = *v*_33_/(*v*_9_ + *v*_22_ + *v*_33_ + *v*_34_). The determined linear system of mass balances, flux ratios, quantitative physiology data, and biomass requirements was then solved with the *fmincon* function from MatLab using the Netto module from FiatFlux to obtain net metabolic fluxes [49].

### Preparation of cell-free extracts and in vitro enzymatic assays

Cell-free extracts were prepared from cells harvested by centrifugation from an appropriate culture volume at 4,000 × *g* at 4 °C for 10 min. Pellets were suspended in 1 volume of 10 mM phosphate-buffered saline (PBS, pH = 7.5, and previously refrigerated) containing 10 mM 2-mercaptoethanol and centrifuged again. Cells were finally resuspended in 0.3–0.5 volume of the same buffer and sonicated intermittently for 6 min in an ice bath. Sonicated cells were centrifuged at 7,500 × *g* at 4 °C for 30 min, and the total protein concentration in cell extracts was measured by the Bradford method. The activities of Edd, Eda, Glk, Gcd, and Gad were assayed using standard protocols [34, 50]. The activity of Zwf and Gnd was assayed under both saturating and non-saturating, *quasi* in vivo conditions using concentrations reflecting intracellular experimentally determined abundance of cofactors and substrates. For reactions in which more than one enzyme catalyzes the corresponding transformation (e.g., Zwf, represented by three isozymes in *P*. *putida* KT2440), the total activity was calculated. In the latter case, the concentrations of the substrates (experimentally determined in cell-free extracts of glucose-grown KT2440 by means of LC-MS/MS), were 1.2 mM G6P and 2.4 mM 6PG.

### Glutathione quantification

Glutathione (both oxidized and reduced forms) was quantified in cells harvested from 25 mL-culture samples using a modification of the procedure described by Michie et al. [51]. Cells were pelleted by centrifugation (4,000 × *g* at 4 °C for 10 min) and suspended in 2 mL of cold PBS. The cell suspension was mixed 1:1 (v/v) with a freshly prepared 10% (w/v) 5-sulfosalicylic acid solution, incubated on an ice bath for 10 min, and then sonicated. For the measurement of total glutathione, 40 μL of Tris(2-carboxyethyl)phosphine·HCl was added to an equal volume of the cell-free extract. For the measurement of reduced glutathione, 40 μL of H_2_O were used instead. Samples were centrifuged at 15,600 × *g* for 5 min after treatment, and 25 μL of the supernatant fluid was added to 100 μL of a buffer containing 200 mM *N*-ethylmorpholine and 20 mM NaOH. Following the addition of 50 μL of 0.5 N NaOH to this mixture, glutathione was derivatized by addition of 10 μL of 10 mM naphthalene-2,3-dicarboxaldehyde. The mixture was incubated at room temperature for 30 min, and the fluorescence intensity of the resulting naphthalene-2,3-dicarboxaldehyde–glutathione conjugate was measured at an excitation wavelength (λ_excitation_) = 472 nm and an emission wavelength (λ_excitation_) = 528 nm. A standard curve was constructed with known amounts of freshly prepared naphthalene-2,3-dicarboxaldehyde–glutathione conjugate.

### Chemicals and enzymes

[1-^13^C_1_]-Glucose and [6-^13^C_1_]-glucose were purchased from Cambridge Isotope Laboratories Inc. (Tewksbury, MA, USA), and [U-^13^C_6_]-glucose was purchased from Sigma-Aldrich Co. (St. Louis, MO, USA). Other chemicals and enzymes used for in vitro assays were obtained from Sigma-Aldrich Co. and Merck KGaA (Darmstadt, Germany). H_2_O_2_ solutions were freshly prepared from a 30% (w/w) stock solution (Sigma-Aldrich Co.).

### Data analysis and statistics

Unless otherwise indicated, all data are presented as mean values together with standard deviation, obtained from at least three independent experiments. Bacterial growth and physiological parameters were analyzed and plotted using GraphPad Prism 7 (GraphPad Software, San Diego, CA, USA) or Microsoft Excel 2019 (Microsoft Corp., Redmond, WA, USA), and statistical differences were calculated using the Student’s *t* test with Welch’s correction. The Student’s *t* test with Welch’s adjustment and the Games-Howell *post-hoc* test were used to calculate statistical differences for metabolomics and fluxome analyses, using *R* version 3.4.3, and a Bonferroni correction was applied, whenever relevant, to correct for multiple comparisons. Actual *p* values of these statistical comparisons are indicated in the figure legends.

## Results

### Quantitative physiology analysis of *P*. *putida* exposed to sub-lethal doses of oxidative stress

**Fig. 1:**
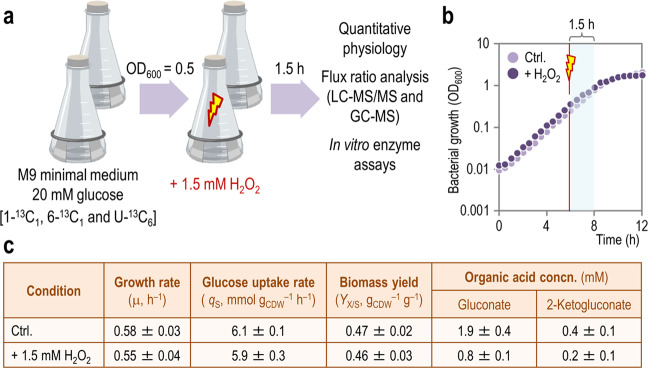
Experimental overview and quantitative physiology parameters for *P*. *putida* KT2440 cultures. **a** Overview of the experimental design adopted in this study. All growth and quantitative physiology experiments were conducted at least in biological triplicates, and the oxidative challenge is indicated with a red symbol. OD_600_, optical density measured at 600 nm. **b** Representative growth curves for control (Ctrl.) experiments and H_2_O_2_-stressed cultures. Samples were taken 1.5 h after addition of H_2_O_2_, where the growth of the cells was still exponential. **c** Key physiological parameters of *P*. *putida* KT2440 in batch glucose cultures. All values represent the mean ±  standard deviations from at least three biological replicates, and the differences between the growth parameters in H_2_O_2_-treated and control cultures was not significant (as assessed by means of the Student’s *t* test with Welch’s correction). The specific growth rate (μ) and the specific rate of carbon uptake (*q*_S_) were determined during exponential growth (including the treatment period) by linear regression of log-transformed OD_600_ data. The yield of biomass on substrate (*Y*_X/S_) was calculated at 24 h, after glucose was completely exhausted. The extracellular concentration (concn.) of organic acids reported is the maximal reached during the whole culture period. CDW, cell dry weight.

In order to capture the metabolic effects of oxidative stress in *P*. *putida* KT2440 without compromising cell viability, the experimental design (summarized in Fig. 1a) entailed exposure of the cells, grown in M9 minimal medium with glucose until mid-exponential phase [optical density at 600 nm (OD_600_) of ca. 0.5], to H_2_O_2_ at 1.5 mM. Cultures with different ^13^C-tracers ([1-^13^C_1_]-, [6-^13^C_1_]- and [U-^13^C_6_]-glucose, all of them added at 20 mM) were grown in parallel to resolve the relative contribution of cyclic EDEMP, PP, and ED pathway to sugar processing. At 1.5 h after the treatment, samples were collected to assess quantitative physiology parameters, flux ratio analysis, and to perform in vitro biochemical assays. This experimental setup resulted in similar growth patterns in the absence and presence of the oxidative stress agent (Fig. 1b), and neither the specific growth rate (μ) nor the final cell density (plateauing at ca. 10 h of cultivation) were significantly affected by the addition of H_2_O_2_ to the cultures. Besides the fact that growth was not significantly affected, this experimental setup was chosen to capture metabolic adaptions at the onset of transcriptional responses due to exposure to oxidative conditions [52, 53]. In particular, no significant changes in the level of expression of genes encoding enzymes of the central metabolism had been observed upon exposure of *P*. *putida* KT2440 to H_2_O_2_ in this time frame [52]. Quantitative analysis of physiology parameters confirmed that this was the case (Fig. 1c), with μ, the specific rate of glucose uptake (*q*_S_) and the yield of biomass on substrate (*Y*_X/S_) being essentially the same in control and H_2_O_2_-treated experiments. The formation and secretion of organic acids to the extracellular milieu, a typical feature of glucose-grown *P*. *putida*, decreased significantly in the presence of the stressor. *P*. *putida* typically oxidizes glucose in the periplasm into gluconate and 2-ketogluconate [32], which are then taken up by the cell [54]. The about halved concentrations of the two products of sugar oxidation in H_2_O_2_-treated cultures indicates a decrease in periplasmic oxidation upon exposure to stressful conditions. The concentration of both acids peaked during exponential growth, and the corresponding molar yields of gluconate and 2-ketogluconate on glucose in control experiments were *y*_G/S_ = 0.31 ± 0.09 and *y*_K/S_ = 0.12 ± 0.02 C-mol C-mol^−1^, respectively. In the presence of oxidative stress, *y*_G/S_ and *y*_K/S_ were 0.19 ± 0.08 and 0.05 ± 0.01 C-mol C-mol^−1^, suggestive, again, of a redistribution of metabolic fluxes between oxidative (i.e. glucose → gluconate → 2-ketogluconate) or phosphorylative [i.e. glucose → G6P] processing routes of the carbon source. Since a shift from periplasmic to cytoplasmic glucose oxidation would entail higher NADPH formation, we next looked for direct evidence of increased intracellular fluxes through these pathways.

### PP pathway metabolite pools increase upon exposure of *P*. *putida* to oxidative stress

**Fig. 2:**
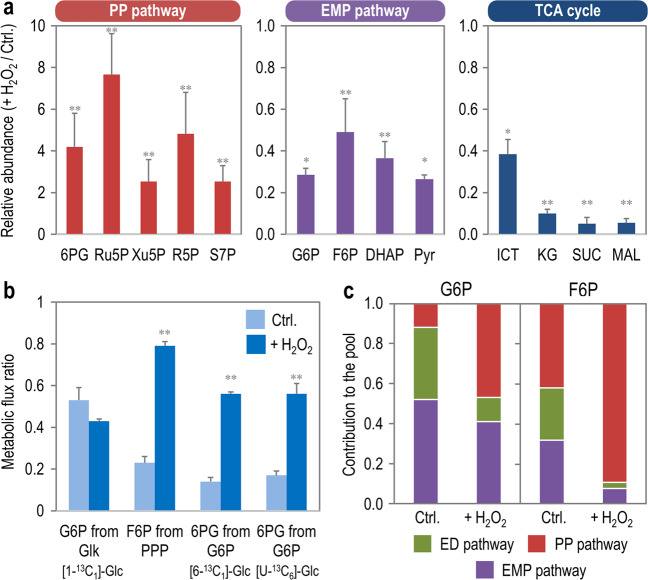
Metabolite levels in *P*. *putida* KT2440 under oxidative stress. **a** Relative abundance of selected metabolites, grouped according to the biochemical block they belong to (i.e. PP pathway, EMP pathway, and TCA cycle). Relative metabolite abundance is expressed as the ratio between H_2_O_2_-induced oxidative stress and control (Ctrl.) conditions, derived from summed ion abundance of all isotopes (counts). Data from experiments in the presence of [1-^13^C_1_]- and [6-^13^C_1_]-glucose (Glc.) are averaged. Bars represent the mean value of metabolite abundance ± standard deviations obtained in triplicate measurements of samples from three independent experiments per labeled substrate. Statistical comparisons between the metabolite abundance ratios (with a ratio = 1 indicating no difference between stressed cultures and control experiments) were assessed by the Student’s *t* test with Welch’s correction. Single (*) and double asterisks (**) identify significant differences at the *p* < 0.05 and *p* < 0.01 levels, respectively. Actual *p* values for the metabolite ratios in the PP pathway (stressed *versus* control experiments) were *p* = 0.0052, 0.0031, 0.0092, 0.0043, and 0.0055, indicated in the same order as the bar graph. For the metabolites in the EMP pathway (stressed *versus* control experiments), the values were *p* = 0.0198, 0.0083, 0.0079, and 0.0412. For the metabolites in the TCA cycle (stressed *versus* control experiments), the values were *p* = 0.0322, 0.0049, 0.0931, and 0.0074. **b** Changes in selected metabolic flux ratios in upper metabolism upon exposure of the cells to H_2_O_2_. The ^13^C-labeled substrate used in each experiment is indicated. Bars represent averages from three independent experiments, and standard deviations were calculated using the covariance matrices of the respective mass distribution vectors by applying the Gaussian law of error propagation. **c** Relative pathway contribution to the G6P and F6P pools. The input of each of the metabolic pathways to the sugar phosphate pool under oxidative stress conditions is indicated with different colors. All abbreviations used in this figure are as indicated in the legend to Fig. S1. CDW, cell dry weight.

To assess the adaptions within the metabolic network of *P*. *putida* upon exposure to oxidative stress, the relative abundance of key metabolites was quantified by LC-MS/MS. Out of the metabolites measured, the species that showed the most significant changes in terms of abundance were intermediates of the PP pathway, the EMP route and the TCA cycle—albeit in opposite directions (Fig. 2a). In particular, the relative abundance of 6PG (key metabolic node where oxidative and phosphorylative pathways for glucose processing converge), R5P, Ru5P, Xu5P, and S7P augmented significantly in H_2_O_2_-stressed cells, ranging from a 2- (Xu5P) to 7.6-fold (Ru5P) increase. At the same time, the relative abundance of glycolytic intermediates of the EMP route decreased roughly by half (e.g., F6P) and up to 70% (e.g., Pyr) when cells were exposed to oxidative stress. Intermediates of the TCA cycle shared the same fate, and their relative abundance was reduced at least by 60% (e.g., isocitrate, ICT) and up to ca. 90% (e.g. α-ketoglutarate, succinate, and malate) in stressed cells. The changes in the relative abundance of other metabolites in the biochemical network of *P*. *putida* KT2440 upon exposure to the oxidative stress agent are listed in Dataset S1. Taken together, these results indicate increased fluxes through the PP pathway coupled to a decrease in the catabolic activities of the EMP route and the TCA cycle during oxidative stress. However suggestive, these metabolomic data cannot be used to infer relative or net fluxes, and flux ratio analysis was employed to piece together the contribution of fluxes within the upper metabolism of *P*. *putida* to the pool of glycolytic intermediates.

### Exposure to oxidative stress increases cyclic operation of PP but not ED pathway

The relative contributions of reverse (i.e., gluconeogenic) flux from triose phosphates or through the PP pathway to the hexose phosphate pool was elucidated by using relative flux ratios derived from [1-^13^C_1_]- and [6-^13^C_1_]-glucose-labeling experiments. Calculation of relative ratios around the hexose node was possible given that the reaction catalyzed by 6-phosphofructo-1-kinase is absent in *P*. *putida* [55, 56], and by considering the reactions mediated by GntZ (6PG dehydrogenase) and Edd (6PG dehydratase) to be irreversible. In particular, using [6-^13^C_1_]-glucose as a tracer enables the resolution of fluxes from the cyclic PP and ED pathways back to hexose phosphates because the C6 position is maintained in both routes and can lead to double-labeled hexose phosphate molecules. Flux ratio analysis supported the notion that fluxes through the PP pathway significantly responded to H_2_O_2_ addition (Fig. 2b), in particular in the oxidative branch of this metabolic pathway.

The metabolic network of *P*. *putida* KT2440 and biochemical reactions considered in this study are summarized in Fig. S1 and Table S1, respectively. Experiments conducted in the presence of [1-^13^C_1_]-glucose suggested that the contribution of glucose kinase (Glk, *v*_3_ in Fig. S1) to the G6P pool was not significantly affected by oxidative stress, whereas the fraction of 6PG from G6P via G6P dehydrogenase (Zwf, *v*_7_ in Fig. S1) increased by at least 3.5-fold (as deduced from experiments conducted with either [6-^13^C_1_]- or [U-^13^C_6_]-glucose) in stressed *P*. *putida* (Fig. 2b). The increase was even more pronounced for the flux ratio reflecting the fraction of F6P from the PP pathway (4.5-fold higher in cultures treated with H_2_O_2_ than in control experiments). The full list of flux ratios, including those for reactions in lower catabolism, is presented in Table S2, and the GC-MS data are given in Dataset S2. Moreover, the contribution of the ED, EMP, and PP routes to the total G6P and F6P pool mirrored the data obtained by flux ratio analysis (Fig. 2c). The PP pathway took a predominant role in feeding the pool of both metabolites when cells were challenged with H_2_O_2_. Taken together, these results provided further evidence for a flux rerouting from periplasmic glucose oxidation to the NADPH-generating PP pathway as a direct metabolic response to oxidative stress. We next quantified net fluxes as the basis for accurate estimation of NADPH production in response to oxidative stress.

### Redistribution of net fluxes in *P*. *putida* as a response to sub-lethal oxidative stress

**Fig. 3:**
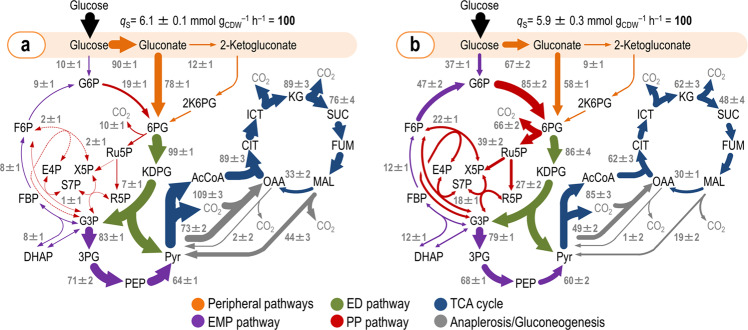
In vivo carbon flux distribution in glucose-grown *P*. *putida* KT2440 obtained from ratio-constrained flux balance analysis. All fluxes, calculated under control conditions (**a**) and in the presence of H_2_O_2_-induced oxidative stress (**b**), were normalized to the specific glucose uptake rate (*q*_S_), and the width of each arrow is scaled to the relative flux. Flux values represent the mean ± standard deviations from at least three biological replicates, after integration of physiological and metabolomics data together with flux ratio analysis. Dashed lines indicate that no significant flux through the corresponding biochemical step was detected under the conditions tested. Abbreviations used for the metabolic intermediates and the main metabolic blocks within the biochemical network are given in the legend to Fig. S1. CDW, cell dry weight.

Combining the quantitative physiology data (Fig. 1c) with the flux ratios as additional constraints (Fig. 2b and Table S2), we estimated net fluxes through the biochemical network (Fig. 3). The values for all the fluxes within the biochemical network are provided in Table S3. Since the relative contribution of the direct phosphorylation of gluconate to 6PG and that of the indirect (NADPH-dependent) route though 2-ketogluconate could not be resolved from ^13^C-labeling data, we determined the in vitro enzyme activities of gluconate kinase (GnuK, i.e. direct phosphorylation of the sugar) and 2-ketogluconate-6-phosphate reductase (KguD, i.e. 6PG from 2-ketogluconate) as a proxy. GnuK and KguD activities in cell-free extracts of *P*. *putida* KT2440 were similar under control and oxidative stress conditions, and we thus used a GnuK/KguD activity ratio of 0.87/0.13 to constrain the flux split at the gluconate branch point.

Under control conditions, glucose was processed mostly through the periplasmic pathway with about 90% of the sugar uptake rate channeled via Gcd (Fig. 3a). Most of the gluconate fed the 6PG node, with a very high flux through the ED route and a ca. 10% recycling of trioses phosphate through the EDEMP cycle. Upon exposure to H_2_O_2_, *P*. *putida* substantially rerouted its carbon fluxes (Fig. 3b). Periplasmic glucose oxidation decreased, and instead much more glucose was imported into the cytoplasm and converted to 6PG through the NADP^+^-dependent Zwf. Since the glucose uptake rate was essentially identical in the two experimental conditions, this large relative flux change reflects a likewise large change in absolute fluxes. In particular, a major change in flux distribution was detected in the non-oxidative branch of the PP pathway. Exposure of the cells to H_2_O_2_ increased the GntZ flux by more than sixfold, which fed the pools of other pentoses phosphate (as suggested by the measurement of their relative abundance increase, Fig. 2). The backward flux in this route fed the F6P and trioses phosphate node, resulting in carbon recycling via glucose-6-phosphate isomerase (Pgi, in the gluconeogenic F6P → G6P direction) and Zwf (G6P → 6PG). Accordingly, fluxes through Pgi and Zwf in H_2_O_2_-treated cells increased by 5.2- and 4.5-fold, respectively. The ED route was still the predominant catabolic pathway for 6PG under oxidative stress, albeit with a slight decrease in the flux values as compared to control conditions. Gluconeogenic fluxes via fructose-1,6-bisphosphate aldolase (Fda) and fructose-1,6-bisphosphatase (Fbp) had a small increase (1.5-fold) in cultures exposed to oxidative stress, and flux through the lower, catabolic branch of the EMP pathway (from glyceraldehyde-3-phosphate to PEP and Pyr) was reduced by ca. 10% under oxidative stress. The diminished input of the ED and the EMP catabolism under oxidative stress propagated into a relatively low flux towards acetyl-CoA (i.e., Pyr dehydrogenase) and fluxes within the TCA cycle. No significant activity of the glyoxylate shunt could be detected under any of the experimental conditions tested. Anaplerotic routes within the Pyr shunt, typical of glucose-grown *P*. *putida*, had a likewise lower activity in H_2_O_2_-stressed cells. The impact of oxidative stress-mediated re-arrangement of carbon fluxes on redox metabolism was investigated next.

### The re-arrangement of net fluxes in *P*. *putida* under oxidative stress increases NADPH supply

**Fig. 4:**
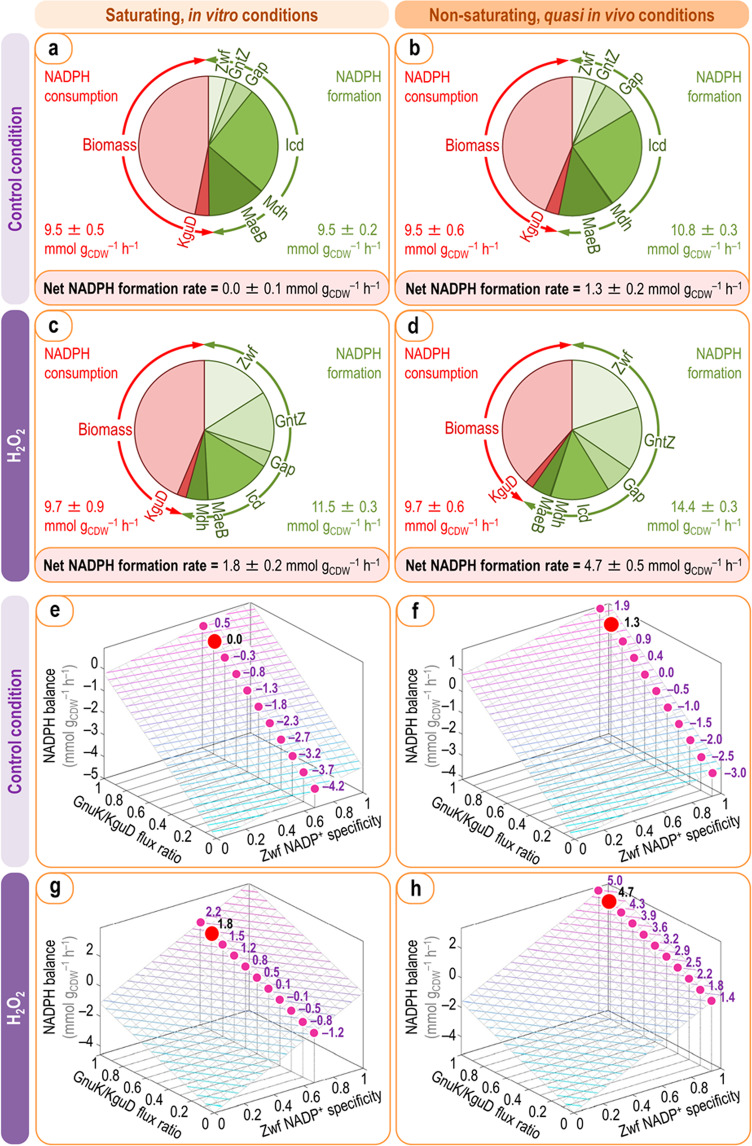
Dynamic NADPH balance in *P*. *putida* KT2440 upon oxidative stress. Overall NADPH balances and sensitivity analysis for the wild-type strain under control (**a**, **b**, **e**, **f**) or H_2_O_2_-induced oxidative stress conditions (**c**, **d**, **g**, **h**) using cofactor specificities of major dehydrogenases under saturating conditions (left column) or *quasi* in vivo conditions (right column). NADPH formation was determined from carbon fluxes through redox cofactor-dependent reactions (Fig. 3 and Table S1) multiplied by experimentally determined relative cofactor specificities. NADPH consumption was calculated from the requirements for biomass production and the actual NADPH-dependent KguD activity. The overall rates of NADPH formation (green) and NADPH consumption (red) are individually indicated (**a**–**d**). Dependence of the overall rates of NADPH turnover on the relative GnuK/KguD flux ratio and the cofactor specificity of Zwf derived from saturating conditions or *quasi* in vivo conditions (**e**–**h**). Actual values for the net NADPH balance are given for the experimentally determined NADP^+^ specificity of Zwf (0.937) as a function of the relative GnuK/KguD flux ratio, with the calculated net rate for each condition indicated with a red dot. CDW, cell dry weight.

Considering that the flux through Zwf and GntZ, two of the major NADP^+^-dependent dehydrogenases in the biochemical network, showed a significant increase under oxidative stress, the overall net NADPH production increased substantially. This reconfiguration presumably matches the increased NADPH demand to regenerate glutathione (and other antioxidants) for H_2_O_2_ reduction. To assess this impact quantitatively, the net rate of NADPH production (*R*_NADPH_) was calculated according to Σ_i_ (*r*_i_^F^ − *r*_i_^C^), where *r*_i_^F^ and *r*_i_^C^ represent the rates of NADPH formation and consumption, respectively, of all major dehydrogenases potentially carrying significant flux in the biochemical network (Fig. S1). Zwf, GntZ, Gap, Icd, Mdh, and MaeB were considered as potential inputs to NADPH formation for this analysis, whereas biomass formation and the KguD activity were included as NADPH sinks. The values of *r*_i_ were derived from the actual previously calculated net fluxes, and the cofactor specificity of all dehydrogenases under both saturating, in vitro conditions and non-saturating, *quasi* in vivo conditions (Table S4) was used to derive the *r*_i_ values. By combining these parameters, *R*_NADPH_ was estimated for *P*. *putida* in both control and H_2_O_2_-treated experiments (Fig. 4). When using cofactor specificities determined under saturating, in vitro conditions, NADPH formation and consumption in control experiments added up (i.e. *R*_NADPH_ = 0, Fig. 4a), whereas adjusting these specificities according to non-saturating, *quasi* in vivo determinations revealed a slight catabolic overproduction of reducing power characteristic of strain KT2440 (*R*_NADPH_ = 1.3 mmol g_CDW_^−1^ h^−1^, Fig. 4b). In H_2_O_2_-stressed cells, *R*_NADPH_ was >0 irrespective of the cofactor specificity used to calculate the individual *r*_i_ values (Fig. 4c, d). In particular, when applying the specificity coefficients derived from non-saturating, more realistic *quasi* in vivo determinations, *R*_NADPH_ increased by 3.6-fold (Fig. 4d) as compared to the control condition (Fig. 4b). Taken together, these results expose a ca. 50% surplus in NADPH formation under oxidative stress conditions (i.e. the flux of NADPH generation increased to 14.4 ± 0.3 mmol g_CDW_^−1^ h^−1^ upon exposure of the cells to H_2_O_2_), that becomes available to counteract ROS accumulation.

To ensure that our NADPH balance estimates are not biased by key assumptions, we assessed the sensitivity of net NADPH formation as a function of the GnuK/KguD activity ratio at the gluconate node and the NADP^+^-specificity of the dehydrogenase reaction catalyzed by Zwf. The Zwf activity of *P*. *putida* KT2440 is represented by three isozymes (i.e. Zwf, ZwfA, and ZwfB; Table S1). Even when we have determined NADP^+^ and NAD^+^ specificities for the overall Zwf reaction with in vitro assays, it is likely that the individual isozymes may have different cofactor preferences, which would have a direct effect on the overall redox balance if expressed differently. Irrespective of the cofactor preferences (0.672 and 0.937 either under saturating conditions or *quasi* in vivo conditions, respectively; see Table S4), *R*_NADPH_ was relatively more responsive to changes in the GnuK/KguD flux ratio in control, non-stressed experiments (Fig. 4e, f). The opposite was true for H_2_O_2_-treated cells, with a strong dependence of *R*_NADPH_ on the output of the Zwf flux (Fig. 4g, h) mainly because the relative flux through Zwf increased in comparison to the control experiment. These calculations demonstrate that the NADPH surplus observed in stressed cells largely stems from the NADP^+^-specificity of Zwf, with the GnuK/KguD flux ratio playing a minor role on *R*_NADPH_ values.

### Altered activities of enzymes involved in glucose catabolism under oxidative stress

**Fig. 5:**
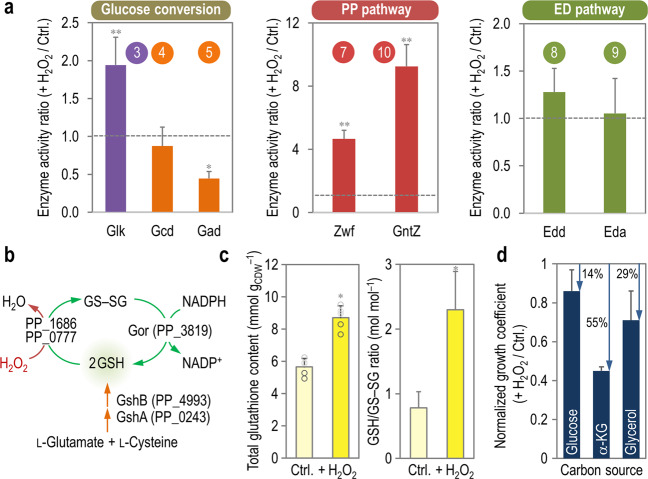
In vitro analysis of key enzymatic activities and glutathione metabolism. **a** Enzyme activity ratios were calculated from the specific activity for each of the indicated reactions assessed under H_2_O_2_-induced oxidative stress and control (Ctrl.) conditions. Each bar represents the mean value of the corresponding ratio ± standard deviations of triplicate measurements from at least two independent experiments, and the horizontal dashed line indicates an activity ratio = 1 (i.e. no changes in enzymatic activities across experimental conditions). Statistical comparisons between enzyme activity ratios were assessed by the Student’s *t* test with Welch’s correction. Single (*) and double asterisks (**) identify significant differences at the *p* < 0.05 and *p* < 0.01 levels, respectively. Actual *p* values for the Glk and Gad activity ratios in the glucose conversion routes were *p* = 0.0019 and 0.0293, respectively. Actual *p* values for the Zwf and GntZ activity ratios in the PP pathway were *p* = 0.0087 and 0.0096, respectively. Circled numbers identify the enzymes in the biochemical network of Fig. S1. **b** Glutathione metabolism in *P*. *putida* KT2440. The key activities involved in biosynthesis and recycling of the reduced (GSH) and oxidized (GS–SG) forms of glutathione are indicated along with the corresponding PP identifiers. **c** Enzymatic determination of total glutathione and the fraction of the oxidized and reduced form. Bars represent the mean value of the corresponding parameter ± standard deviations of duplicate measurements from at least five independent experiments, with individual measurements indicated as empty circles, and the asterisk (*) identifies significant differences between stressed cells and control conditions at *P* < 0.05 as assessed by the Student’s *t* test with the Bonferroni correc*t*ion. Actual *p* values for the total glutathione content and the GSH/GS–SG ratio between H_2_O_2_-treated and control cultures were *p* = 0.0361 and 0.0117, respectively. CDW, cell dry weight. **d** Impact of the carbon substrate on the growth of *P*. *putida* KT2440 upon an oxidative challenge. Normalized growth coefficients, representing the fraction of the specific growth rate (μ) in the presence of 3 mM H_2_O_2_ as compared with that of control (Ctrl.) conditions, were calculated in cultures using glucose, α-ketoglutarate (α-KG) or glycerol as the carbon source. Each bar represents the mean value of the normalized growth coefficients ± standard deviations of triplicate independent experiments, while the arrows and the accompanying percentages indicate the relative reduction in the growth rate under oxidative stress.

To further validate the results of in vivo distribution of metabolic fluxes, in vitro measurements of key enzymatic activities were carried out in cell-free extracts of *P*. *putida* KT2440 incubated under the same conditions. In agreement with the relative flux distribution (Fig. 3), there was a significant decrease in the activities of enzymes within the peripheral reactions for glucose oxidation under oxidative stress conditions (Fig. 5a). The observed decrease was particularly noticeable in the case of the specific Gad activity (gluconate → 2-ketogluconate), which was roughly halved in cells exposed to H_2_O_2_. The hexose kinase activity, accordingly, increased by twofold. Changes in the activity of the two NADPH producing dehydrogenases of the PP pathway were more prominent, with the specific Zwf and GntZ activities increasing by 4.7- and 9.2-fold under oxidative stress, respectively. The in vitro activities of two enzymes of the ED pathway, in contrast, remained essentially unchanged within the experimental error in both control and H_2_O_2_-stressed cells. Although we cannot rule out that gene expression (and thus, protein levels) could have been affected in the time frame of the experiments, the results of the in vitro enzyme determinations are fully consistent with the previous observations in terms of metabolic flux distribution. Taken together, these results underline the importance of PP pathway for NADPH production when cells are challenged with an oxidative stress agent. We next explored the connection between catabolic NADPH overproduction and mechanisms that counteract oxidative stress such as the glutathione-dependent redox system.

### The glutathione system of *P*. *putida* links H_2_O_2_-induced overproduction of NADPH to ROS quenching in a carbon source-dependent fashion

Thiols play several roles in bacteria, which includes maintaining the redox balance, quenching ROS and nitrogen reactive species, and detoxifying other toxins and stress-inducing factors [57]. In most organisms, the major thiol involved in such processes is the tripeptide glutathione (GSH, γ–L-glutamyl-L-cysteinylglycine). The formation of GSH from L-glutamate and L-cysteine in strain KT2440 is catalyzed by GshAB (glutamate-cysteine ligase and glutathione synthase, respectively; Fig. 5b). The glutathione cycle, connecting the reduced (GSH) and the oxidized (GS–SG) forms of the thiol, acts as a first-line reductant of ROS (Fig. 5b) via glutathione peroxidases (e.g., PP_0777 and PP_1686) and glutaredoxins. Glutathione reductase (Gor, PP_3819) then replenishes the GSH pool by using NADPH as the reducing currency. Since the glutathione system of *P*. *putida* has not been explored thus far from a biochemical point of view, the cellular content of both GSH and GS–SG was assessed by means of a biochemical assay in both control and H_2_O_2_-stressed cultures (Fig. 5c). Cells exposed to H_2_O_2_ had a 1.6-fold higher total content of glutathione (i.e. GSH and GS–SG) than control experiments, and the composition of the total pool changed significantly upon exposure to oxidative stress. The GSH/GS–SG molar ratio, which reflects the fraction of reduced thiol in the cells, increased by threefold in H_2_O_2_–stressed *P*. *putida*. This observation suggests that the reduced form of glutathione can be sufficiently replenished by the cellular NADPH sources upon an oxidative stress—directly linking the increased NADPH turnover with the pool of antioxidants.

Considering that there is a direct connection between the carbon source on the overall metabolic regime adopted by *P*. *putida*—and thus, the operation of the EDEMP cycle—we explored oxidative stress tolerance when using substrates processed through mainly glycolytic, gluconeogenic, and mixed biochemical routes (Fig. 5d). To this end, the bacterial growth of cultures developed on the presence of glucose, α-ketoglutarate or glycerol was monitored upon an oxidative challenge by adding H_2_O_2_ at 3 mM (i.e., twice the amount used in the experiments above, and thereby affecting growth rates). While the growth of *P*. *putida* with glycolytic substrates, which foster NADPH-forming routes as explained above, was not significantly affected under stressful conditions (with a reduction of μ of only 14%), the other substrates promoted higher levels of sensitivity. The use of a mostly gluconeogenic substrate (α-ketoglutarate) led to μ values in cultures added with H_2_O_2_ of around half those observed in control experiments. Glycerol, which is partially processed by gluconeogenic and glycolytic modules of central metabolism [58], promoted a level of tolerance to oxidative stress in between the two other substrates (Fig. 5d). Although growth phenotypes are the consequence of several layers of multi-component regulation, these results expose the connection between the operativity of different segments of the versatile metabolism of strain KT2440, the coarse NADPH supply and the level of tolerance to oxidative stress.

## Discussion

Environmental microorganisms are exposed to a variety of physicochemical perturbations that require dynamic and concerted responses to ensure cell survival [59]. On this background, *P*. *putida* KT2440 provides a unique experimental system to explore the interplay between stress and metabolic adaption. Broadening this understanding has not only a fundamental interest, but also practical consequences in view of the growing demand of robust agents for in situ environmental catalysis [60] and metabolic engineering [61]. In actively growing bacterial cells, metabolic fluxes are adapted to meet energy and redox demands [62], and a trade-off between steady-state growth rates and physiological adaptability in fluctuating environments seems to be a conserved feature of growing bacteria [63]. The distribution of metabolic fluxes under conditions where growth is not affected, on the other hand, is a largely unexplored aspect. Environmental bacteria do not divide actively in the natural niches they colonize, yet the cells are exposed to stressful conditions under these near-zero growth state—as mimicked in our current experimental design. Moreover, exploring nongrowing conditions has a significant impact on the design of industrial biocatalysts and bioprocesses [64], where stressful conditions are prevalent—and *P*. *putida* KT2440 is naturally primed to endow such circumstances [65].

The results of the present study strengthen the notion that network-wide adaption of metabolic fluxes [62, 66] accompanies (and plausibly, enables) the phenomenon traditionally described—at a genetic and regulatory level—as the *general stress response* [52, 53, 67, 68]. Metabolic adaption to oxidative stress has been revisited by Christodoulou et al. [30], demonstrating that reserve flux capacity in the PP pathway empowers *E*. *coli* to rapidly respond to H_2_O_2_ by increased flux through Zwf and Gnd. It was postulated that inhibition of Zwf caused by NADPH (either by a competitive, allosteric or combined mechanism) is lifted under these conditions—releasing ‘reserve’ flux through the oxidative PP pathway. Moreover, the activity of glyceraldehyde-3-phosphate is lowered due to ROS-mediated damage, which causes a metabolic *jam* in the metabolite pools of the PP route, as previously shown in redox-stressed yeast [69]. Product inhibition of Zwf is relieved due to NADPH drainage to counteract oxidative stress [70], and the suddenly enlarged 6PG pool is now channeled via (NADPH-forming) Gnd. Recently, such an NADPH replenishing mechanism catalyzed by Zwf and Gnd has been recognized as a general strategy in enteric bacteria, yeast, and mammalian cells [71]. In this study, we asked how *P*. *putida*, which lacks a linear, EMP-based glycolysis and processes glucose mostly through the conversion of the sugar into gluconate, reacts to oxidative stress. Since glycolysis is not an alternative glucose degradation route (therefore, *P. putida* cannot alter the split ratio between the EMP and PP pathways), we hypothesized that the EDEMP cycle could contribute to increased NADPH biosynthesis under oxidative stress via the PP pathway branch.

**Fig. 6:**
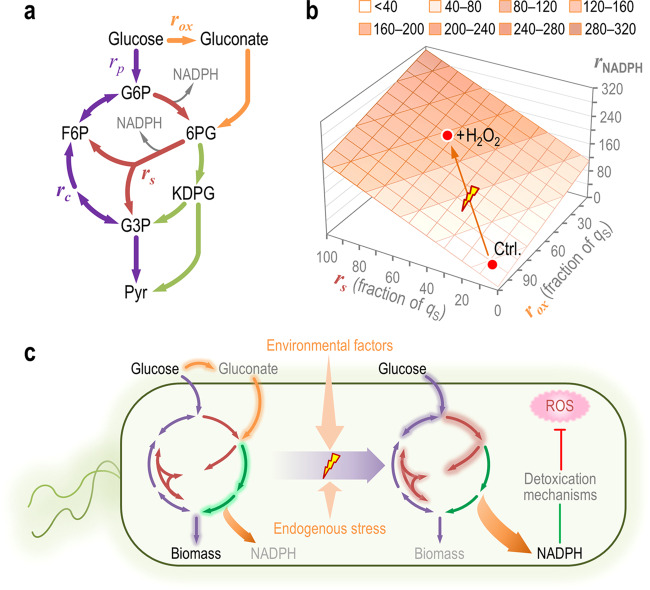
The architecture of central carbon metabolism in *P*. *putida* enables rapid supply of NADPH upon oxidative stress. **a** Schematic representation of the upper metabolism of *P*. *putida* KT2440. Several biochemical reaction have been lumped to illustrate the main routes for carbon circulation (see Fig. S1 for details and abbreviations). Note that the total rate of carbon uptake (*q*_S_) is split between glucose phosphorylation and oxidation to gluconate, such that *q*_S_ = *r*_*p*_ + *r*_*ox*_. The overall cycling flux of trioses phosphate towards hexoses phosphate is indicated as *r*_*c*_ and the flux through the PP pathway shunt is termed *r*_*s*_. Cofactors other than NADPH have been omitted in the drawing for the sake of clarity. **b** Functional relationship between *r*_NADPH_, the rate of NADPH formation within the simplified metabolic network of (**a**), and the fluxes through the oxidative loop for glucose processing and the PP pathway shunt. All (arbitrary) values are given as a fraction of *q*_S_, and the experimental conditions tested in this work are indicated with red dots (Ctrl. control conditions). **c** General model for flux distribution in the upper metabolic domain of *P*. *putida*. Under normal growth conditions, glucose is processed mostly through its oxidative conversion to gluconate, and the EDEMP cycle provides intermediates for biomass, with a very low flux through the PP pathway. Upon oxidative stress conditions (exerted either by endogenous or external perturbations), a rapid increase of fluxes via the PP pathway shunt replenishes the intermediates within upper metabolism and provides a direct source of NADPH that can be coupled to anti-oxidant defense mechanisms against reactive oxygen species (ROS). Fluxes predominant under each condition are highlighted.

Metabolite changes and flux analysis revealed that the lower (EMP) glycolysis and the PP pathway were affected by H_2_O_2_ as compared with untreated controls. In a simplified model of the upper pathways for sugar processing (Fig. 6a), the output of NADPH is largely governed by the fluxes through Zwf and GntZ (Gnd)—that, in turn, respond to the split between oxidative conversion of glucose into gluconate or phosphorylation into G6P, and the recycling activity of the EDEMP cycle. As such, the overall NADPH turnover is dependent on *r*_*s*_ (Fig. 6b), further modulated by the fraction of glucose oxidized in the bacterial periplasm. Consequently, a low activity of the EDEMP cycle contributes to NADPH formation when cells grow in the absence of any oxidative stress. Upon an oxidative challenge, the backward flux from the PP and ED routes to F6P increases >3-fold, and the ED flux through lower glycolysis decreases due to re-arrangement. The boost in the activities through the cyclic PP pathway is most remarkable, as *P*. *putida* maintains very low fluxes within this metabolic block under normal growth conditions [72]. Such metabolic adaption mechanism entails an increase in the fluxes through the enzymes needed for cyclic operation that burn carbon into CO_2_ to generate NADPH. This alteration of activities that lead to NADPH formation could stem (at least partially) from de novo transcriptional rearrangements—although the expression level of genes encoding the main NADP^+^-dependent dehydrogenases in the central catabolism of *P*. *putida* KT2440 seem not be overly affected by the H_2_O_2_ treatment [52]. One way or the other, under these conditions the overall biomass yield is similar to that observed in control, non-stressed cultures because formation of CO_2_ by mechanisms connected to cellular respiration (i.e. pyruvate dehydrogenase and the TCA cycle) is reduced. *P*. *putida* deploys a survival strategy that seems to differ from that reported for the close relative species *P*. *fluorescens*, where production of α-ketoacids and ATP formation via converging metabolic mechanisms prevails under oxidative stress [68, 73]—yet the observed responses are dependent on the carbon substrates used for both species.

Increasing evidence points to glutathione peroxidase as a key survival mechanism in stressed bacteria [74–76]. Glutathione peroxidase-dependent reduction of ROS requires a continuous supply of NADPH for regeneration [77]. Upon oxidative stress, the glutathione-based detoxification of ROS drains a lot of NADPH that must be replenished via the mechanisms explained above. Importantly, the results of this study reflect a situation where the immediate, early metabolic response (which in *E*. *coli* takes place within the first 10 s after exposure to the stress agent) is blended with early transcriptional responses (e.g., activation of inducible defense mechanisms, which take several minutes to occur). Experimental evidence indicates that there is a multilayered array of defense strategies against oxidative stress in *P*. *putida*, including (i) several catalases and superoxide dismutases [78, 79], (ii) ferredoxin-NADP^+^ oxido-reductases [80], (iii) polyphosphate-dependent NAD^+^ and NADH kinases [81], and (iv) stress-induced transhydrogenation activities [44]. However important, these mechanisms can be classified as (relatively) late responses to oxidative insults as they mostly depend on transcriptional regulation, and the re-arrangement of metabolic fluxes to ensure NADPH supply appears to represent a first line of defense in several bacterial species [82].

Regardless of which carbon source is used, the EDEMP cycle forms the metabolic core of *P. putida*, and virtually every anabolic or catabolic transaction of the cell involves the operation of this metabolic architecture. The reallocation of metabolic resources for the sake of generating redox currency NADPH under stressful conditions is not an exclusive feature of glucose-dependent growth (Fig. S1 and Table S1). On the contrary, the EDEMP cycle is the major biochemical device that endows this species with the metabolic versatility needed to tolerate redox stress independently of the main nutrients at hand. Moreover, the data presented above accounts to a large extent for the ability of *P. putida* [83] to withstand a variety of environmental insults—not just oxidative conditions. Desiccation and osmotic pressure [84], organic solvents [85], starvation [86], radiation [87] (including sunlight [88]), high temperature [89], and other physicochemical and nutritional circumstances [90] that often prevail in natural niches typically converge toward producing ROS. This is a well-documented phenomenon in the case of antibiotic treatment: the ensuing, indirectly produced ROS seem to be the ultimate agent of bacterial death [91] beyond the primary action on basic cellular functions. Any mechanism that counteracts ROS may thus result in shifting the survival window of the bacterium towards a more hostile environment, as it seems to be the case for *P. putida*. In either circumstance, an abundant, conditional NADPH supply is a key feature to ensure cell survival and durability in changing environmental (and industrial) scenarios. Given the capability of *P*. *putida* to adapt to stressful conditions, it cannot come as a surprise that this species has received considerable attention in recent years as a biological agent of choice for in situ bioremediation [60] in a time of global pollution challenges [92].

## Supplementary information

Supplementary Information

Data Set 1

Data Set 2

## Data Availability

Metabolite levels and ^13^C-labeling data are available in the Supplementary Information files. Other datasets generated and analyzed in this study are available from the corresponding authors on request.
