# Supplementary material for: Reconfiguration of metabolic fluxes in Pseudomonas putida as a response to sub-lethal oxidative stress
Source: ISME J. 2021 Jan 11;15(6):1751–66. doi: 10.1038/s41396-020-00884-9 (PMC8163872; doi:10.1038/s41396-020-00884-9)
Supplement: Supplementary file 1 — Supplementary Information [file 41396_2020_884_MOESM1_ESM.pdf]

Supplementary Information

Reconfiguration of metabolic fluxes in *Pseudomonas putida* as a response to sub-lethal oxidative stress

Pablo I. Nikel, Tobias Fuhrer, Max Chavarría, Alberto Sánchez-Pascuala, Uwe Sauer, and Víctor de Lorenzo

---

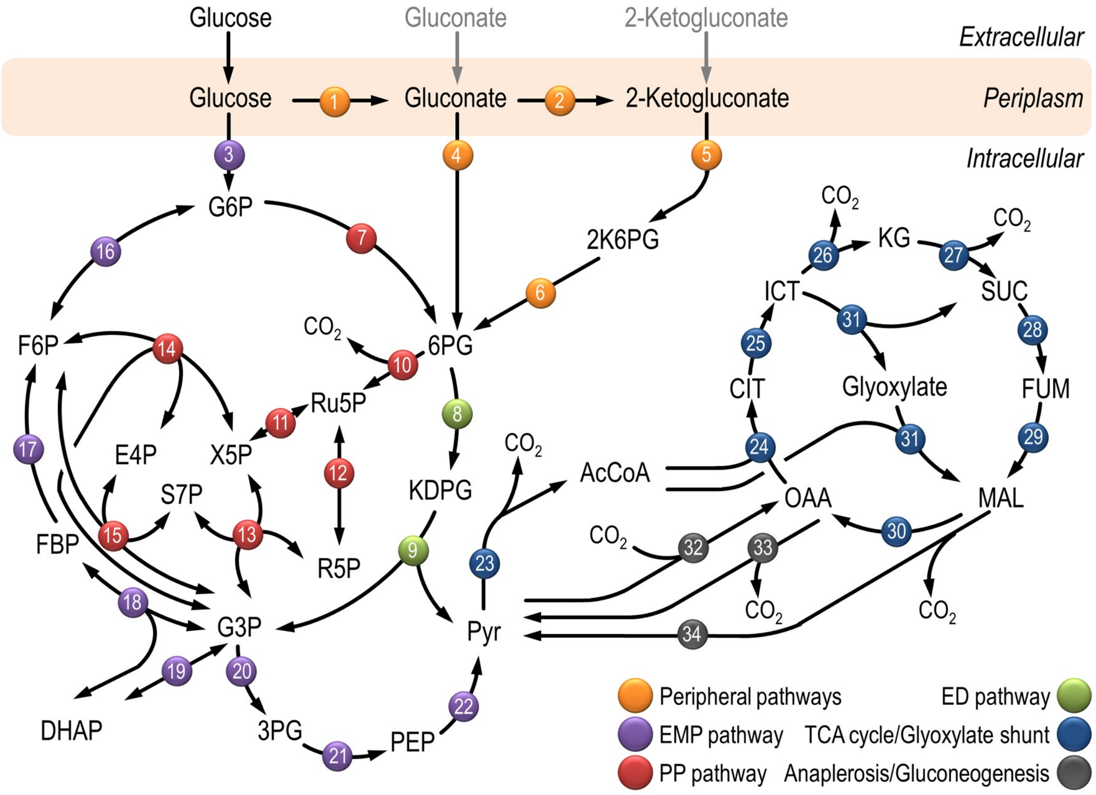

Figure S1. Biochemical pathways involved in glucose catabolism in *Pseudomonas putida* KT2440. The metabolic network is sketched around six main metabolic blocks, identified with different colors: [i] peripheral pathways, i.e. oxidative transformation of glucose into gluconate and 2-ketogluconate (and their corresponding phosphorylated derivatives); [ii] Embden-Meyerhof-Parnas (EMP) pathway (non-functional, since 6-phosphofructo-1-kinase is absent); [iii] pentose phosphate (PP) pathway; [iv] Entner-Doudoroff (ED) pathway; [v] tricarboxylic acid (TCA) cycle and glyoxylate shunt; and [vi] anaplerotic and gluconeogenic reactions. The oxidative transformations in the outer membrane and in the periplasmic space are shown at the top of the scheme, along with the transport systems for glucose, gluconate and 2-ketogluconate into the cytoplasm. Some reactions have been lumped to simplify the diagram. Transport of gluconate and 2-ketogluconate from the extracellular space is indicated by gray arrows. All the reactions in the network have been assigned a number (circled), and the complete list of the enzymes and isozymes catalyzing each step is listed in **Table S1**. The reaction catalyzed by the pyruvate dehydrogenase complex (v_{23}) was arbitrarily assigned to the TCA cycle and glyoxylate shunt block for the sake of simplicity, lumping together all metabolites downstream pyruvate. Abbreviations are as follows: G6P, glucose-6-phosphate; F6P, fructose-6-phosphate; FBP, fructose-1,6-bisphosphate; DHAP, dihydroxyacetone phosphate; 6PG, 6-phosphogluconate; KDPG, 2-keto-3-deoxy-6-phosphogluconate; 2K6PG, 2-keto-6-phosphogluconate; Ru5P, ribulose-5-phosphate; R5P, ribose-5-phosphate; X5P, xylulose-5-phosphate; S7P, sedoheptulose-7-phosphate; E4P, erythrose-4-phosphate; G3P, glyceraldehyde-3-phosphate; 3PG, 3-phosphoglycerate; PEP, phosphoenolpyruvate; Pyr, pyruvate; AcCoA, acetyl-coenzyme A; OAA, oxaloacetate; CIT, citrate; ICT, isocitrate; KG, 2-ketoglutarate; SUC, succinate; FUM, fumarate; and MAL, malate.

Table S1. Components of the biochemical network of *Pseudomonas putida* KT2440[a].

Block	Code	Reaction	Enzyme(s)	Name(s) and PP number(s)
Peripheral pathways	1	Glucose + Ubiquinone → Glucono-1,5-lactone + Ubiquinol	Glucose dehydrogenase	Gcd (PP_1444)
		Glucono-1,5-lactone + H_2O → Gluconate + H^+	Gluconolactonase	Gnl (PP_1170)
	2	Gluconate + Ubiquinone → 2-Ketogluconate + Ubiquinol	Gluconate 2-dehydrogenase	PP_3382 PP_3383 PP_3384
	4	Gluconate + ATP → 6PG + ADP + H^+	Gluconate kinase	GnuK (PP_3416)
	5	2-Ketogluconate + ATP → 2K6PG + ADP + H^+	2-Ketoglucokinase	KguK (PP_3378)
	6	2K6PG + NADPH + H^+ → 6PG + $NADP^+$	2-Ketogluconate-6-phosphate reductase	KguD (PP_3376)
Pentose phosphate pathway	7	G6P + $NADP^+$ → 6-Phosphoglucono-1,5-lactone + NADPH + H^+	Glucose-6-phosphate 1-dehydrogenase	ZwfA (PP_1022) ZwfB (PP_4042) Zwf (PP_5351)
		6-Phosphoglucono-1,5-lactone + H_2O → 6PG + H^+	6-Phospho-gluconolactonase	Pgl (PP_1023)
	10	6PG + $NADP^+$ → Ru5P + NADPH + CO_2	6-Phosphogluconate dehydrogenase	GntZ (PP_4043)
	11	Ru5P ↔ Xu5P	Ribulose-5-phosphate 3-epimerase	Rpe (PP_0415)
	12	Ru5P ↔ Ri5P	Ribose-5-phosphate isomerase	RpiA (PP_5150)
	13	Xu5P + R5P ↔ S7P + G3P	Transketolase	TktA (PP_4965)
	14	Xu5P + E4P ↔ G3P + F6P		
	15	S7P + G3P ↔ E4P + F6P	Transaldolase	Tal (PP_2168)
Entner-Doudoroff pathway	8	6PG → KDPG + H_2O	6-Phosphogluconate dehydratase	Edd (PP_1010)
	9	KDPG → G3P + Pyr	2-Keto-3-deoxy-6-phosphogluconate aldolase	Eda (PP_1024)

Embden-Meyerhof-Parnas pathway	3	Glucose + ATP → G6P + ADP + H^+	Glucokinase	Glk (PP_1011)
	16	G6P → F6P	Glucose-6-phosphate isomerase	Pgi-I (PP_1808) Pgi-II (PP_4701)
	17	FBP + H_2O + ADP → F6P + ATP	Fructose-1,6-bisphosphatase	Fbp (PP_5040)
	18	DHAP + G3P ↔ FBP	Fructose-1,6-bisphosphate aldolase	Fda (PP_4960) PP_2871 PP_3224
	19	G3P ↔ DHAP	Triose phosphate isomerase	TpiA (PP_4715)
	20	G3P + NAD^+ + Pi → 1,3-Bisphosphoglycerate + NADH + H^+	Glyceraldehyde-3-phosphate dehydrogenase	GapA (PP_1009) GapB (PP_2149) PP_0665 PP_3443
		1,3-Bisphosphoglycerate + ADP → 3PG + ATP	Phosphoglycerate kinase	Pgk (PP_4963)
	21	3PG → 2PG	Phosphoglycerate mutase	GpmI (PP_5056) PP_2243 PP_3923 PP_4450
		2PG → PEP + H_2O	Enolase	Eno (PP_1612)
	22	PEP + ADP + H^+ → Pyr + ATP	Pyruvate kinase	PykA (PP_1362) Pyk (PP_4301)
Tricarboxylic acid cycle / Glyoxylate shunt	23	Pyr + NAD^+ + Coenzyme A → AcCoA + NADH + CO_2	Pyruvate dehydrogenase	AcoA (PP_0555) AcoC (PP_0553) AceF (PP_0338) AceE (PP_0339)
	24	OAA + AcCoA + H_2O → CIT + Coenzyme A + H^+	Citrate synthase	GltA (PP_4194)
	25	CIT → ICT	Aconitate hydratase	AcnA-I (PP_2112) AcnA-II (PP_2336) AcnB (PP_2339)

Tricarboxylic acid cycle / Glyoxylate shunt	26	ICT + $NADP^+$ → KG + CO_2 + NADPH + H^+	Isocitrate dehydrogenase	Icd (PP_4011) Idh (PP_4012)
	27	KG + Coenzyme A + NAD^+ → Succinyl-Coenzyme A + NADH + H^+ + CO_2	2-Ketoglutarate dehydrogenase	Lpd (PP_5366) LpdG (PP_4187) LpdV (PP_4404) SucA (PP_4189) SucB (PP_4188) PP2652 PP3662
		Succinyl-Coenzyme A + ADP + Pi → SUC + Coenzyme A + ATP	Succinyl-coenzyme A synthetase	SucC (PP_4186) SucD (PP_4185) ScpC (PP_0154)
	28	SUC + Ubiquinone → FUM + Ubiquinol	Succinate dehydrogenase	SdhA (PP_4191) SdhB (PP_4190) SdhC (PP_4193) SdhD (PP_4192)
	29	FUM + H_2O → MAL	Fumarate hydratase	FumC-I (PP_0944) FumC-II (PP_1755) PP_0897
	30	MAL + NAD^+ (quinone) → OAA + NADH (quinol) + H^+	Malate dehydrogenase / Malate:quinone oxidoreductase	Mdh (PP_0654) PP_3591 Mqo-1 (PP_0751) Mqo-2 (PP_1251) Mqo-3 (PP2925)
	31	ICT → SUC + Glyoxylate	Isocitrate lyase	AceA (PP_4116)
		Glyoxylate + AcCoA + H_2O → MAL + Coenzyme A + H^+	Malate synthase	GlcB (PP_0356)

Anaplerosis / Gluconeogenesis	32	Pyr + $CO_2 \rightarrow$ OAA + H^+	Pyruvate carboxylase	PycA (PP_5347) PycB (PP_5346)
	33	OAA + Pi $\rightarrow$ PEP + CO_2	Phosphoenol-pyruvate carboxylase[d]	Ppc (PP_1505)
	34	MAL + $NADP^+ \rightarrow$ Pyr + CO_2 + NADPH	Malic enzyme	MaeB (PP_5085)

[a] Information compiled from the *Pseudomonas* Genome Database [1, 2], MetaCyc [3] and the literature [4-6]. In the instances in which no gene name has been assigned, the PP number is given for each open reading frame. Biochemical reactions are coded according to the six functional blocks indicated in **Fig. S1**. Note that the reaction catalyzed by the pyruvate dehydrogenase complex (v_{23}) has been arbitrarily assigned to the TCA cycle for the sake of simplicity. According to Nelson *et al*. [7], *pckA* (*PP_0253*, encoding phosphoenolpyruvate carboxykinase) contains an authentic frameshift and therefore the open reading frame is classified as a pseudogene in the *Pseudomonas* Genome Database. All abbreviations are defined in the legend to **Fig. S1**. Pi, inorganic orthophosphate.

Table S2. Selected metabolic flux ratios used for ratio-constrained flux balance analysis[a].

Metabolic flux ratio	Code(s) in **Fig. S1**	Ratio (mean ± SD)[e]	
		Control conditions	+ H_2O_2
G6P from glucose[b]	3	0.53 ± 0.06	0.43 ± 0.01
6PG from G6P[b]	7	0.14 ± 0.02	0.56 ± 0.01
6PG from G6P[c]	7	0.17 ± 0.02	0.56 ± 0.05
F6P from the PP pathway[c]	14, 15	0.23 ± 0.03	0.79 ± 0.02
Pyruvate through the ED pathway[b]	9	0.44 ± 0.02	0.51 ± 0.03
Glyoxylate shunt[d]	31	N.D.	N.D.
OAA from pyuvate[d]	32	0.65 ± 0.06	0.63 ± 0.06
Phosphoenolpyruvate from oxaloacetate[d]	33	0.00 ± 0.04	0.01 ± 0.04
Pyruvate from malate (UB)[d]	34	0.71 ± 0.16	0.65 ± 0.14
Pyruvate from malate (LB)[d]	34	0.25 ± 0.04	0.24 ± 0.03

[a] Flux ratios are shaded according to the metabolic block they belong according to **Fig. S1** and **Table S1**. Abbreviations: G6P, glucose-6-phosphate; 6PG, 6-phosphogluconate; F6P, fructose-6-phosphate; PP pathway, pentose phosphate pathway; ED pathway, Entner-Doudoroff pathway; UB, upper bound; LB, lower bound; SD, standard deviation; and ND, not detected.

[b] Determined from 100% [1-^{13}C]-glucose experiments.

[c] Determined from 100% [6-^{13}C]-glucose experiments.

[d] Determined from 20% [U-$^{13}C_6$]-glucose experiments.

[e] Standard deviations (SD) for each relative metabolic flux ratio were calculated using the covariance matrices of the respective mass distribution vectors by applying the Gaussian law of error propagation.

Table S3. Net flux values for central metabolic pathways[a] of *P. putida* KT2440 grown on glucose under control and oxidative stress conditions.

Functional block	Reaction	**Flux value** (mmol g_{CDW}^{-1} h^{-1}) **± standard error**	
		**Control conditions**	**+ H_2O_2**
Peripheral pathways	1	5.53 ± 0.05	3.96 ± 0.07
	2	0.72 ± 0.04	0.52 ± 0.04
	4	4.79 ± 0.07	3.45 ± 0.07
	5	0.72 ± 0.04	0.52 ± 0.04
	6	0.72 ± 0.04	0.52 ± 0.04
Pentose phosphate pathway	7	1.17 ± 0.08	5.02 ± 0.05
	10	0.59 ± 0.03	3.89 ± 0.13
	11	0.15 ± 0.59	2.31 ± 3.89
	12	0.44 ± 0.59	1.58 ± 3.89
	13	0.15 ± 0.01	1.28 ± 0.05
	14	0.01 ± 0.01	1.04 ± 0.05
	15	0.15 ± 0.01	1.28 ± 0.05
Entner-Doudoroff pathway	8	6.12 ± 0.05	5.09 ± 0.05
	9	6.12 ± 0.05	5.09 ± 0.05
Embden-Meyerhof-Parnas pathway	3	0.63 ± 0.04	2.18 ± 0.07
	16	0.57 ± 0.04	2.87 ± 0.11
	17	0.46 ± 0.03	0.61 ± 0.04
	18	0.46 ± 0.03	0.61 ± 0.04
	19	0.46 ± 0.03	0.61 ± 0.04
	20	5.11 ± 0.04	4.86 ± 0.05
	21	4.36 ± 0.07	4.03 ± 0.07
	22	3.89 ± 0.08	3.53 ± 0.08
Tricarboxylic acid cycle / Glyoxylate shunt	23	6.67 ± 0.18	5.01 ± 0.17
	24	5.44 ± 0.21	3.67 ± 0.18
	25	5.44 ± 0.21	3.67 ± 0.18
	26	5.44 ± 0.21	3.67 ± 0.18
	27	4.71 ± 0.22	2.85 ± 0.21
	28	4.71 ± 0.22	2.85 ± 0.21
	29	4.71 ± 0.22	2.85 ± 0.21
	30	2.01 ± 0.11	1.75 ± 0.09
	31	0.00 ± 0.00	0.00 ± 0.00
Anaplerosis / Gluconeogenesis	32	4.44 ± 0.15	2.89 ± 0.12
	33	0.15 ± 0.13	0.04 ± 0.11
	34	2.69 ± 0.18	1.12 ± 0.15

[a] The classification and codes of the biochemical reactions is the same as depicted in **Fig. S1** and **Table S1** in the Supplementary Information. The distribution of normalized values for each metabolic flux normalized to the specific rate of glucose consumption is shown in **Fig. 3** in the main text.

Table S4. Cofactor specificity for the main dehydrogenases in the central metabolism of *Pseudomonas putida* KT2440.

Enzyme	Enzyme(s)	Relative cofactor specificity (%) under			
		Saturating conditions		Non-saturating, *quasi in vivo* conditions	
		NAD^+	$NADP^+$	NAD^+	$NADP^+$
G6P dehydrogenase	ZwfA (PP_1022)<br>ZwfB (PP_4042)<br>Zwf (PP_5351)	32.8 ± 3.6	67.2 ± 9.5	6.3 ± 0.5	93.7 ± 1.1
6PG dehydrogenase	GntZ (PP_4043)	23.9 ± 0.8	76.1 ± 3.2	8.6 ± 0.7	91.4 ± 1.2
ICT dehydrogenase	Icd (PP_4011)<br>Idh (PP_4012)	11.8 ± 1.3	88.2 ± 3.3	11.1 ± 0.5	88.9 ± 2.6
MAL dehydrogenase	Mdh (PP_0654)<br>PP_5391	98.4 ± 1.1	1.6 ± 0.3	97.5 ± 2.9	2.5 ± 0.9
2K6PG reductase	KguD (PP_3376)	13.3 ± 0.5	86.7 ± 1.3	10.2 ± 0.8	89.8 ± 1.3
G3P dehydrogenase	GapA (PP_1009)<br>GapB (PP_2149)<br>PP_0665<br>PP_3443	84.1 ± 7.2	15.9 ± 1.3	66.8 ± 0.9	33.2 ± 0.1
Malic enzyme	MaeB (PP_5085)	4.6 ± 1.6	95.4 ± 2.4	3.6 ± 0.1	96.4 ± 2.7

[a] Values represent the mean of the relative cofactor specificity ± standard deviation of triplicate measurements from at least two independent experiments conducted in the presence of either NAD($^+$/H) or NADP($^+$/H). All the enzymatic activities were assayed in cell-free extracts obtained from exponentially-growing cells cultured on M9 minimal medium containing 20 mM glucose. In the case of activities represented by more than one enzyme, the cofactor specificity of the total activity is given.

Description of other supplementary datasets

Dataset 1. Raw ^{13}C-labelling data. Raw intensities in counts for metabolites measured (0 = M_0 isotope, 1 = M + 1, etc.) in two biological replicates. Excel file.

Dataset 2. Raw GC-MS data. GC-MS mass distribution vectors (MDV) were corrected for natural abundance from 20% [U-$^{13}C_6$]-glucose experiments. In the data, –15, –57, –85 and f309 represent the different fragments of derivatized amino acids with the respective fractional abundance for the isotopes M_0, M + 1, …, M_{max}. Relative flux ratios are averages from four biological replicates. Excel file.

REFERENCES

1. Winsor GL, Lam DKW, Fleming L, Lo R, Whiteside MD, Yu NY, et al. *Pseudomonas* Genome Database: improved comparative analysis and population genomics capability for *Pseudomonas* genomes. Nucleic Acids Res. 2011;39:D596-D600.
2. Winsor GL, Griffiths EJ, Lo R, Dhillon BK, Shay JA, Brinkman FS. Enhanced annotations and features for comparing thousands of *Pseudomonas* genomes in the *Pseudomonas* Genome Database. Nucleic Acids Res. 2016;44:D646-D53.
3. Caspi R, Billington R, Fulcher CA, Keseler IM, Kothari A, Krummenacker M, et al. The MetaCyc database of metabolic pathways and enzymes. Nucleic Acids Res. 2018;46:D633-D9.
4. Nikel PI, Martínez-García E, de Lorenzo V. Biotechnological domestication of pseudomonads using synthetic biology. Nat Rev Microbiol. 2014;12:368-79.
5. Nikel PI, Chavarría M, Fuhrer T, Sauer U, de Lorenzo V. *Pseudomonas putida* KT2440 strain metabolizes glucose through a cycle formed by enzymes of the Entner-Doudoroff, Embden-Meyerhof-Parnas, and pentose phosphate pathways. J Biol Chem. 2015;290:25920-32.
6. Nogales J, Mueller J, Gudmundsson S, Canalejo FJ, Duque E, Monk J, et al. High-quality genome-scale metabolic modelling of *Pseudomonas putida* highlights its broad metabolic capabilities. Environ Microbiol. 2020;22:255-69.
7. Nelson KE, Weinel C, Paulsen IT, Dodson RJ, Hilbert H, Martins dos Santos VAP, et al. Complete genome sequence and comparative analysis of the metabolically versatile *Pseudomonas putida* KT2440. Environ Microbiol. 2002;4:799-808.
